# Effect of pericentric inversion of chromosome 9 on reproductive outcomes in assisted reproductive technology: a propensity score–matched cohort study

**DOI:** 10.3389/fendo.2026.1811641

**Published:** 2026-03-26

**Authors:** Wenjie Huang, Xiaoping Ren, Huihui Xu, Jiaoyan Liu, Liqiong Duan, Yanhuan Ou, Weiyou Lv, Fengying Zhang, Li Fan, Cunmei Su, Huawei Wang

**Affiliations:** 1 Reproductive Genetics Department, The First Affiliated Hospital of Kunming Medical University, Kunming, Yunnan, China; 2 Department of Reproductive Medicine, Guangzhou Women and Children’s Medical Center Liuzhou Hospital, Liuzhou, China; 3 Department of Reproductive Medicine, Liuzhou Maternity and Child Healthcare Hospital, Liuzhou, China

**Keywords:** assisted reproductive technology, chromosome 9 inversion, *in vitro* fertilization, propensity score matching, reproductive outcomes

## Abstract

**Background:**

Pericentric inversion of chromosome 9 [inv (9)] is a common chromosomal polymorphism found in approximately 1-2% of the population. While inv(9) has been generally considered a benign variant, its potential effects on reproductive outcomes, especially in the context of assisted reproductive technology (ART), remain unclear. This study aimed to evaluate whether inv(9) affects embryologic and reproductive outcomes in ART cycles.

**Methods:**

This retrospective cohort study included 40,502 ART cycles conducted between September 2008 to December 2023, of which 903 involved couples carrying inv(9) and 39,599 involved couples with normal karyotypes (NK). Propensity score matching (PSM) was used to balance baseline characteristics. The primary outcome was live birth rate (LBR), and secondary outcomes included biochemical and clinical pregnancy, miscarriage, and neonatal outcomes among live births (multiple births and low birth weight), and cycle parameters (oocyte yield, fertilization, and blastocyst formation rates).

**Results:**

After 1:2 PSM, baseline characteristics were balanced between inv(9) and NK groups. Cycle outcomes, including oocytes retrieved (10.0 ± 7.8 vs 9.9 ± 7.5), fertilization, and blastocyst formation rates, were comparable. In fresh embryo transfer (ET) cycles, inv(9) carriers had a modestly higher live birth rate compared to NK couples (46.4% vs 41.2%; aRR = 1.13; 95% CI 1.01–1.27), while no significant differences were observed in frozen embryo transfer (FET) cycles (41.1% vs 40.0%; aRR = 1.04; 95% CI 0.93–1.17). Other pregnancy outcomes (multiple births, low birth weight, biochemical pregnancy, clinical pregnancy, and miscarriage) were similar between groups. No significant differences in live birth, clinical pregnancy, miscarriage, multiple births, or low birth weight were observed between male and female inv(9) carriers in either fresh or frozen cycles.

**Conclusions:**

Pericentric inversion of chromosome 9 was not associated with adverse embryologic or reproductive outcomes in ART. These findings support the classification of inv(9) as a benign chromosomal variant and provide reassurance that its presence does not warrant additional intervention in couples undergoing ART.

## Introduction

In modern assisted reproductive technology (ART), the ultimate goal for patients extends beyond merely achieving embryo transfer (ET); rather, it encompasses attaining a healthy live birth while minimizing the risk of complications (1). Among the many challenges encountered in ART, genetic counseling has become an increasingly critical and time-consuming step, particularly when chromosomal abnormalities are detected. This process often requires a multidisciplinary approach involving reproductive physicians, genetic counselors, and embryologists, aiming to provide patients with a comprehensive interpretation of genetic findings. Importantly, counseling profoundly influences couples’ decisions throughout the treatment process, ultimately shaping their reproductive choices and success rates (2).

From a genetic perspective, chromosomal polymorphisms—structural variations typically occurring in heterochromatic regions of chromosomes 1, 9, and 16, the distal Y chromosome, and the short arms or satellites of D- and G-group chromosomes (13–15, 21, 22)—constitute an important factor potentially influencing ART outcomes, with chromosome 9 inversion [inv (9)] being one of the most frequent variants in humans (3–5). The prevalence of inv (9) varies among ethnic groups, ranging from approximately 0.26% to 3.57% in the general population, with reported rates of 1%–2% in Asian populations and a slightly higher frequency in females (6).

The potential reproductive consequences of inv (9) have been the focus of considerable research, yet the findings remain inconsistent. Several studies have examined associations between inv (9) and impaired sperm quality (7, 8), sperm abnormalities (9), embryo aneuploidy (10, 11), intrauterine fetal demise (12), and recurrent miscarriage (5, 13, 14). However, despite these investigations, evidence remains scarce and inconclusive regarding how inv (9) carriage in either partner affects embryo development, pregnancy outcomes, or neonatal health in the ART setting (15, 16). As ART continues to evolve and genetic screening becomes increasingly widespread, clarifying the clinical implications of inv (9) has become particularly important. Understanding whether inv (9) impacts embryologic or reproductive outcomes could provide valuable insights for pre-treatment counseling and prognostic assessment among affected couples.

Therefore, the present study aimed to comprehensively evaluate the influence of inv (9) on ART outcomes, including embryologic parameters, pregnancy success, and live birth rates, by analyzing a large cohort of cycles with propensity score–matched controls. By comparing couples carrying inv (9) with those exhibiting normal karyotypes (NK), we sought to determine whether this common chromosomal polymorphism exerts any measurable effect on reproductive performance or clinical outcomes in the ART setting. Findings from this study may offer meaningful evidence to guide genetic counseling and optimize clinical management for couples undergoing ART.

## Materials and methods

### Study design and setting

We performed a retrospective cohort analysis at the Reproductive Medicine Center of Guangzhou Women and Children’s Medical Center Liuzhou Hospital. The study period spanned September 2008 through December 2023. The protocol was approved by the institutional ethics committee (approval number: 2025-147). Because de-identified clinical data were used without any intervention, informed consent was waived.

### Study population and eligibility

Consecutive couples undergoing autologous IVF/ICSI during the study period were screened. Eligibility required: (a) both partners completed peripheral blood karyotyping before treatment with G-banding resolution ≥320–400 bands and results available for review; and (b) at least one oocyte retrieval (for embryological outcomes) or at least one embryo transfer—fresh or frozen (for pregnancy/live-birth outcomes).

We excluded cycles if either partner had any additional structural or numerical chromosomal abnormality beyond inversion 9, including reciprocal/Robertsonian translocations, inversions other than 9, deletions/duplications/additions, ring or marker chromosomes, or mosaic aneuploidies (e.g., 45,X; 47,XXY; 45,X/46,XX). Common heteromorphic variants (e.g., 1/9/16qh±, D/G group ps/pss/pstk/sat variants, Yqh±) were excluded. Cycles using donor oocytes, donor sperm, dual-donor, or gestational carriers were excluded. Cycles were also excluded if key outcomes were missing or if karyotype reports were uninterpretable. Couples in whom both partners carried inversion 9 were rare (n=4) and were excluded *a priori* from comparative analyses. A flow diagram illustrating study selection and propensity score matching is provided in **Supplementary Figure S1**.

### Exposure

Karyotypes were categorized as: 1) NK (normal karyotype): both partners 46,XX/46,XY; 2) F-inv (9): female partner with chromosome 9 inversion, male partner NK; 3) M-inv (9): male partner with chromosome 9 inversion, female partner NK.

The primary comparison contrasted inv (9) carriers (combined) vs NK; prespecified subgroup analyses contrasted M-inv (9) vs F-inv (9).

### Clinical care, laboratory procedures, and embryo transfer

Detailed clinical and laboratory protocols at our center have been reported previously (17). Briefly, ovarian stimulation, monitoring, ovulation trigger, and oocyte retrieval followed routine institutional protocols. Regimens included GnRH agonist, GnRH antagonist, luteal-phase stimulation, progestin-primed ovarian stimulation, mild stimulation, and natural-cycle approaches, with gonadotropin dosing titrated to follicular response and serum hormones. Fertilization was by conventional IVF or ICSI per clinical indication. Embryos were cultured in bench-top incubators under controlled gas and temperature, with routine assessment of fertilization and daily morphologic evaluation to the cleavage or blastocyst stage. Fresh ETs were performed on day 3 or day 5/6 according to embryo development and endometrial readiness. For FET, the endometrium was prepared with either natural or programmed regimens, and transfers were performed under ultrasound guidance per institutional policy.

### Outcomes

The primary outcome was live birth, defined as delivery of ≥1 live-born infant. Secondary pregnancy outcomes included biochemical pregnancy (a positive serum β-hCG test without ultrasound confirmation of an intrauterine gestational sac), clinical pregnancy (intrauterine gestational sac on ultrasound or documented outcome), miscarriage (spontaneous loss <20 gestational weeks), multiple births (delivery of ≥2 live-born infants in a single pregnancy), and low birth weight (neonatal birth weight <2500 g). Embryological outcomes included counts of oocytes retrieved, mature (MII) oocytes, fertilized oocytes, good-quality embryos, and blastocysts, as well as maturation, fertilization, and blastocyst-formation rates.

### Propensity score matching and covariates

Analyses were conducted at the cycle level, allowing multiple cycles per couple. To mitigate baseline differences, we implemented 1:2 PSM comparing inv (9) to NK cycles. Propensity scores were derived from pre-treatment demographic and clinical variables: female age, BMI, AMH, basal E2/FSH/LH, total gonadotropin dose, infertility duration, infertility type, infertility diagnosis, fertilization method, and ovarian stimulation protocol. To account for secular trends in clinical practice and laboratory performance, calendar time of oocyte retrieval was explicitly incorporated into the matching set (i.e., the matching algorithm was constrained by retrieval time). Covariate balance was evaluated using standardized mean differences (SMDs), with |SMD| ≥0.10 indicating meaningful imbalance. For transfer outcomes, we performed independent 1:2 PSM within the fresh ET and FET cohorts, forcing the embryo-transfer date and using transfer-specific covariates (e.g., gravidity/parity, number of embryos transferred, endometrial thickness, transfer day; for FET, endometrial preparation in lieu of stimulation protocol).

### Statistical analysis

All analyses were conducted using R software (version 4.3.0), and two-sided P values <0.05 were considered statistically significant. Continuous variables were summarized as mean ± SD and compared using the t-test or Wilcoxon rank-sum test as appropriate. Categorical variables were expressed as counts and percentages and compared using the χ² or Fisher’s exact test.

To minimize baseline differences between couples carrying inv (9) and those with NK, PSM was applied using nearest-neighbor 1:2 matching without replacement. Missing values (<5% for most variables) were considered missing at random and imputed using the median (for continuous variables) or mode (for categorical variables).

All primary and secondary outcome analyses were conducted at the cycle level. Because individual couples could contribute more than one ART cycle, we accounted for within-couple correlation by using modified Poisson regression models with cluster-robust (sandwich) standard errors, clustering on the unique patient identifier (representing the couple), to estimate adjusted relative risks (aRRs) and 95% confidence intervals (CIs) for binary outcomes (e.g., live birth, multiple births, low birth weight, biochemical pregnancy, clinical pregnancy, and miscarriage).

For count outcomes (e.g., number of oocytes retrieved, mature oocytes, and blastocysts formed), Poisson regression with cluster-robust standard errors was used to estimate adjusted mean ratios (ratios of expected counts; exponentiated coefficients) and 95% CIs. Linear regression models with cluster-robust variance were applied for continuous or proportional outcomes (e.g., maturation, fertilization, and blastocyst formation rates).

Subgroup analyses compared male vs. female inv (9) carriers, and interaction analyses examined potential effect modification by baseline factors (e.g., age, AMH, BMI, infertility type, and gonadotropin dose).

## Results

A total of 40,502 ART cycles were analyzed, including 903 (2.2%) cycles from couples carrying an inv (9) and 39,599 (97.8%) cycles from couples with NK. After 1:2 PSM based on clinical characteristics and oocyte retrieval time, 903 inv (9) cycles were matched to 1,806 NK cycles with adequate covariate balance (**Table 1**). Before matching, couples with inv (9) showed marginally higher AMH levels and basal LH values, whereas other demographic and clinical parameters were largely comparable. After matching, most baseline variables were well balanced, with SMDs below 0.1; a slight residual imbalance remained for infertility diagnosis (SMD = 0.115), which was further adjusted for in subsequent analyses. The mean female age was approximately 34 years, and the mean BMI was around 22 kg/m² in both groups. The distribution of infertility type, fertilization method, and ovarian stimulation protocol was similar between NK and inv (9) couples. Among inv (9) carriers, 401 (44.4%) involved M-inv (9) and 502 (55.6%) involved F-inv (9) (**Supplementary Table S1**). The two subgroups were comparable in most parameters, although M-inv (9) couples had a longer duration of infertility (5.50 ± 4.61 vs 4.74 ± 4.08 years, P = 0.01).

**Table 1:** Baseline characteristics of couples with normal karyotypes and those with inversion of chromosome 9 before and after 1:2 propensity score matching.

Characteristic	Overall, n = 40502	Before matching			After matching		
		NK = 39599	inv(9) = 903	SMD	NK = 1806	inv(9) = 903	SMD
Age	34.51 ± 5.40	34.52 ± 5.41	34.29 ± 5.16	0.043	34.18 ± 5.51	34.29 ± 5.16	0.021
AMH (ng/mL)	2.71 ± 2.57	2.71 ± 2.57	2.84 ± 2.60	0.049	2.92 ± 2.73	2.84 ± 2.60	0.032
BMI (kg/m^2^)	22.07 ± 3.10	22.07 ± 3.09	22.13 ± 3.33	0.019	22.17 ± 3.09	22.13 ± 3.33	0.012
Basal E2 (pg/mL)	50.03 ± 87.15	50.05 ± 87.61	49.03 ± 63.81	0.013	50.13 ± 66.96	49.03 ± 63.81	0.017
Basal FSH (mIU/mL)	7.21 ± 7.66	7.21 ± 7.73	7.19 ± 3.98	0.003	7.19 ± 5.30	7.19 ± 3.98	<0.001
Basal LH (mIU/mL)	3.96 ± 3.25	3.95 ± 3.23	4.15 ± 4.07	0.055	4.30 ± 4.20	4.15 ± 4.07	0.037
Total Gn dose (IU)	1762.0 ± 904.1	1762.5 ± 904.2	1739.6 ± 898.2	0.025	1748.5 ± 899.3	1739.6 ± 898.2	0.010
Infertility duration (y)	4.94 ± 4.09	4.94 ± 4.09	5.07 ± 4.34	0.032	4.97 ± 4.17	5.07 ± 4.34	0.023
Fertilization method (%)				0.010			0.002
IVF	29415 (72.6)	28763 (72.6)	652 (72.2)		1302 (72.1)	652 (72.2)	
ICSI	11087 (27.4)	10836 (27.4)	251 (27.8)		504 (27.9)	251 (27.8)	
Infertility type (%)				0.027			0.015
Primary	13749 (33.9)	13431 (33.9)	318 (35.2)		649 (35.9)	318 (35.2)	
Secondary	26753 (66.1)	26168 (66.1)	585 (64.8)		1157 (64.1)	585 (64.8)	
Infertility diagnosis (%)				0.134			0.115
Tubal factor	25742 (63.6)	25165 (63.5)	577 (63.9)		1183 (65.5)	577 (63.9)	
Male factor	5166 (12.8)	5059 (12.8)	107 (11.8)		223 (12.3)	107 (11.8)	
Ovulatory	2769 (6.8)	2688 (6.8)	81 (9.0)		125 (6.9)	81 (9.0)	
Genetic factor	1334 (3.3)	1300 (3.3)	34 (3.8)		60 (3.3)	34 (3.8)	
RPL	790 (2.0)	768 (1.9)	22 (2.4)		42 (2.3)	22 (2.4)	
Endometriosis	1146 (2.8)	1130 (2.9)	16 (1.8)		43 (2.4)	16 (1.8)	
Unknown	2731 (6.7)	2684 (6.8)	47 (5.2)		107 (5.9)	47 (5.2)	
Other	824 (2.0)	805 (2.0)	19 (2.1)		23 (1.3)	19 (2.1)	
Ovarian stimulation protocol, No. (%)				0.046			0.073
Agonist	23221 (57.3)	22707 (57.3)	514 (56.9)		1017 (56.3)	514 (56.9)	
Antagonist	8621 (21.3)	8425 (21.3)	196 (21.7)		406 (22.5)	196 (21.7)	
Luteal-phase stimulation	893 (2.2)	869 (2.2)	24 (2.7)		35 (1.9)	24 (2.7)	
PPOS	1953 (4.8)	1906 (4.8)	47 (5.2)		103 (5.7)	47 (5.2)	
Mild Stimulation	4229 (10.4)	4139 (10.5)	90 (10.0)		194 (10.7)	90 (10.0)	
Natural cycles	595 (1.5)	582 (1.5)	13 (1.4)		21 (1.2)	13 (1.4)	
Other	990 (2.4)	971 (2.5)	19 (2.1)		30 (1.7)	19 (2.1)	

Continuous variables are presented as mean ± standard deviation (SD), and categorical variables are shown as number (percentage). Data are reported before and after 1:2 propensity score matching (PSM). The standardized mean difference (SMD) was used to assess covariate balance between groups, with an absolute SMD ≥ 0.10 indicating a meaningful imbalance. BMI, body mass index (calculated as weight in kilograms divided by height in meters squared); SD, standard deviation; PSM, propensity score matching; SMD, standardized mean difference; AMH, anti-Müllerian hormone; E_2_, estradiol; FSH, follicle-stimulating hormone; LH, luteinizing hormone; Gn, gonadotropin; IVF, *in vitro* fertilization; ICSI, intracytoplasmic sperm injection; PPOS, progestin-primed ovarian stimulation; RPL, recurrent pregnancy loss.

a Percentages may not total 100% because of rounding.

b Infertility diagnosis categories (only the predominant diagnosis was recorded when multiple causes coexisted): “tubal factor” refers to the fallopian tubes being blocked or damaged; “male factor” refers to reduced sperm concentrations or other abnormalities in sperm function that impede fertilization under normal conditions; “ovulatory dysfunction” refers to irregular or absent ovulation; “genetic factor” includes chromosomal abnormalities such as structural rearrangements; “recurrent pregnancy loss (RPL)” was defined as two or more consecutive miscarriages; “endometriosis” refers to the presence of endometrial-like tissue outside the uterine cavity; “unknown” indicates cases with no clear etiology after standard evaluation; and “other” includes diagnoses that do not meet criteria for any other category.

c Infertility type was categorized as primary (no prior conception) or secondary (at least one previous conception).

d Ovarian stimulation protocols included agonist, antagonist, luteal-phase stimulation, progestin-primed ovarian stimulation (PPOS), mild stimulation, natural cycles, and other regimens.

In the subset of fresh ET cycles, 21,661 cycles were included, comprising 472 (2.2%) from inv (9) couples and 21,189 (97.8%) from NK couples. Following 1:2 PSM, 472 inv (9) cycles were matched to 944 NK cycles (**Supplementary Table S3**). Prior to matching, inv (9) couples exhibited slightly lower parity and received smaller gonadotropin doses; these differences diminished after matching, resulting in balanced groups (all SMDs < 0.1). The two cohorts showed comparable distributions in age, BMI, ovarian response, fertilization method, and endometrial parameters. Within the inv (9) subset, 207 cycles were from M-inv (9) and 265 from F-inv (9) carriers (**Supplementary Table S4**). Baseline features were overall similar, except for higher BMI in F-inv (9) women (22.50 ± 3.73 vs 21.88 ± 3.05 kg/m²; P = 0.046) and a higher proportion of day 5/6 transfers (26.0% vs 19.3%).

Among FET cycles, 28,522 were analyzed, with 623 (2.2%) inv (9) and 27,899 (97.8%) NK cycles. After matching, 623 inv (9) cycles were compared with 1,246 NK cycles (**Supplementary Table S5**). Before matching, couples carrying inv (9) had slightly higher BMI and longer infertility duration; these differences were eliminated after PSM, yielding balanced groups (SMDs < 0.1). Baseline characteristics such as female age, AMH, and endometrial thickness were similar between groups. Within the inv (9) cohort, 264 cycles involved M-inv (9) and 359 involved F-inv (9) (**Supplementary Table S6**). Compared with M-inv (9) couples, F-inv (9) couples showed lower BMI (21.81 ± 3.29 vs 22.39 ± 3.23 kg/m²; P = 0.028) and shorter infertility duration (4.89 ± 3.97 vs 5.67 ± 4.83 years; P = 0.033), while other clinical variables were comparable.

### Embryological and cycle outcomes

Following propensity score matching, cycle characteristics were comparable between couples with NK and those carrying inv (9) (**Table 2**). The mean numbers of oocytes retrieved (10.0 ± 7.8 vs 9.9 ± 7.5), mature (MII) oocytes (8.9 ± 7.0 vs 8.8 ± 6.7), fertilized oocytes (6.4 ± 5.3 vs 6.3 ± 5.2), and blastocysts formed (4.9 ± 3.7 vs 4.9 ± 3.8) were nearly identical between NK and inv (9) groups, with no statistically significant differences in adjusted models (adjusted mean ratios close to 1.0, P > 0.05). Similarly, maturation, fertilization, and blastocyst formation rates were comparable between groups (all β values ≈ 0, P > 0.05). When stratified by carrier sex, couples with M-inv (9) and F-inv (9) showed comparable cycle outcomes (**Table 3**). Although the F-inv (9) group demonstrated numerically higher counts of good-quality embryos (3.3 ± 3.7 vs 2.9 ± 3.0) and blastocysts (5.1 ± 4.1 vs 4.7 ± 3.6), these differences were not statistically significant. Combined analyses including NK, M-inv (9), and F-inv (9) cohorts confirmed similar distributions across all embryologic parameters (**Supplementary Table S2**).

**Table 2:** Cycle outcomes after propensity score matching.

Cycle characteristics	NK = 1806	inv(9) = 903
Oocytes retrieved	10.0 ± 7.8	9.9 ± 7.5
	Ref (1.0)	1.009 (0.961-1.059)
Mature oocytes	8.9 ± 7.0	8.8 ± 6.7
	Ref (1.0)	1.005 (0.955-1.057)
Total fertilized	6.4 ± 5.3	6.3 ± 5.2
	Ref (1.0)	1.000 (0.945-1.058)
Good-quality embryos	3.2 ± 3.4	3.2 ± 3.4
	Ref (1.0)	1.005 (0.926-1.090)
Blastocyst cultured	8.6 ± 5.2	8.5 ± 5.2
	Ref (1.0)	0.985 (0.928-1.047)
Blastocyst formation	4.9 ± 3.7	4.9 ± 3.8
	Ref (1.0)	0.993 (0.919-1.071)
Mature rate (per oocyte retrieved, %)^a^	87.2 ± 19.1	87.1 ± 18.6
	Ref (1.0)	-0.21 (-1.85-1.42)
Fertilization rate (2PN, per oocyte retrieved, %)^a^	63.5 ± 25.5	64.0 ± 24.5
	Ref (1.0)	0.12 (-1.97-2.21)
Blastocyst formation rate (per blastocyst cultured, %)^a^	55.6 ± 26.0	57.3 ± 26.9
	Ref (1.0)	1.08 (-1.74-3.90)

Values are expressed as mean ± standard deviation (SD). Poisson regression models with a log link and cluster-robust standard errors clustered by patient ID were used to estimate adjusted mean ratios and 95% CIs, accounting for potential confounders including female age, BMI, AMH, E2, FSH, LH, total gonadotropin dose, infertility duration, fertilization method, infertility type, infertility diagnosis, and ovarian stimulation protocol in the matched cohort.

a Linear regression models were applied to continuous percentage outcomes (e.g., mature rate, fertilization rate, and blastocyst formation rate) to estimate regression coefficients (β) and 95% CIs, adjusting for the same covariates.

**Table 3:** Cycle outcomes by sex of inv(9) carrier.

Cycle characteristics	M-inv(9) = 401	F-inv(9) = 502
Oocytes retrieved	9.8 ± 7.4	10.0 ± 7.5
	Ref (1.0)	1.004 (0.932-1.082)
Mature oocytes	8.6 ± 6.4	8.9 ± 6.9
	Ref (1.0)	1.024 (0.948-1.106)
Total fertilized	6.1 ± 4.8	6.4 ± 5.5
	Ref (1.0)	1.039 (0.951-1.135)
Good-quality embryos	2.9 ± 3.0	3.3 ± 3.7
	Ref (1.0)	1.129 (0.990-1.287)
Blastocyst cultured	8.1 ± 4.7	8.8 ± 5.5
	Ref (1.0)	1.073 (0.973-1.182)
Blastocyst formation	4.7 ± 3.6	5.1 ± 4.1
	Ref (1.0)	1.097 (0.966-1.245)
Mature rate (per oocyte retrieved, %)	86.6 ± 19.5	87.4 ± 17.9
	Ref (1.0)	0.71 (-1.76-3.17)
Fertilization rate (2PN, per oocyte retrieved, %)	64.2 ± 24.4	63.8 ± 24.5
	Ref (1.0)	-0.33 (-3.61-2.95)
Blastocyst formation rate (per blastocyst cultured, %)	57.1 ± 27.5	57.5 ± 26.4
	Ref (1.0)	0.63 (-3.99-5.25)

Values are expressed as mean ± standard deviation (SD).

Poisson regression models with a log link and cluster-robust standard errors clustered by patient ID were used to estimate adjusted mean ratios (95% CI), and linear regression models were applied for continuous percentage outcomes to estimate regression coefficients (β) (95% CI). All models were adjusted for the same covariates as in **Table 2**.

### Pregnancy outcomes

In fresh ET cycles, the live birth rate (LBR)—the primary outcome—was modestly higher among inv (9) couples compared with NK (46.4% vs 41.2%; aRR = 1.13; 95% CI 1.01–1.27; **Table 4**). Other pregnancy outcomes were comparable between groups, including multiple births among live births (18.3% vs 17.0%; aRR = 0.942; 95% CI 0.669–1.328) and low birth weight among live births (20.5% vs 18.0%; aRR = 1.058; 95% CI 0.755–1.481), as well as biochemical pregnancy, clinical pregnancy, and miscarriage. In FET cycles, multiple births among live births (7.8% vs 12.4%; aRR = 0.731; 95% CI 0.461–1.160) and low birth weight among live births (14.5% vs 14.0%; aRR = 1.078; 95% CI 0.750–1.549) were also similar between groups (**Supplementary Figure S2**).

**Table 4:** Pregnancy outcomes after fresh and frozen embryo transfer.

Outcomes	NK group (n, %)	inv(9) group (n, %)	Effect estimate (95% CI)	
			Unadjusted	Multivariable adjusted
Live birth (Primary outcome)	389/944 (41.2%)	219/472 (46.4%)	1.126 (0.994-1.275)	1.130 (1.006-1.270)^a^
Multiple births	66/389 (17.0%)	40/219 (18.3%)	1.077 (0.754-1.537)	0.942 (0.669-1.328)
Low birth weight	70/389 (18.0%)	45/219 (20.5%)	1.142 (0.816-1.598)	1.058 (0.755-1.481)
Biochemical pregnancy	519/944 (55.0%)	267/472 (56.6%)	1.029 (0.933-1.135)	1.030 (0.938-1.131)
Clinical pregnancy	476/944 (50.4%)	248/472 (52.5%)	1.042 (0.936-1.160)	1.044 (0.943-1.155)
Miscarriage	75/944 (7.9%)	27/472 (5.7%)	0.720 (0.468-1.108)	0.732 (0.476-1.125)
FET				
Live birth (Primary outcome)	499/1246 (40.0%)	256/623 (41.1%)	1.026 (0.909-1.158)	1.044 (0.932-1.170)^b^
Multiple births	62/499 (12.4%)	20/256 (7.8%)	0.629 (0.389-1.017)	0.731 (0.461-1.160)
Low birth weight	70/499 (14.0%)	37/256 (14.5%)	1.030 (0.712-1.490)	1.078 (0.750-1.549)
Biochemical pregnancy	683/1246 (54.8%)	346/623 (55.5%)	1.013 (0.924-1.111)	1.026 (0.941-1.119)
Clinical pregnancy	621/1246 (49.8%)	324/623 (52.0%)	1.043 (0.944-1.153)	1.058 (0.963-1.164)
Miscarriage	108/1246 (8.7%)	60/623 (9.6%)	1.111 (0.813-1.518)	1.126 (0.827-1.534)

Data are presented as number (percentage). Modified Poisson regression with a log link was used to estimate adjusted risk ratios (aRRs) and 95% confidence intervals (CIs) for all binary outcomes. Cluster-robust standard errors were applied, with clustering by patient identifier to account for within-patient correlation across multiple embryo transfer cycles. For neonatal outcomes, multiple births and low birth weight were calculated among live births, with live births as the denominator.

a Fresh cycle models were adjusted for female age, BMI, AMH, infertility duration, gravidity, parity, total gonadotropin dose, number of embryos transferred, endometrial thickness, fertilization method, infertility type and diagnosis, transfer day, and ovarian stimulation protocol.

b Models for FET cycles were adjusted for female age, BMI, AMH, infertility duration, gravidity, parity, number of embryos transferred, endometrial thickness, fertilization method, type of infertility, infertility diagnosis, day of embryo transfer, and endometrial preparation regimen.

Among inv (9) carriers, pregnancy outcomes were comparable between M-inv (9) and F-inv (9) in both fresh and frozen cycles, including live birth, and neonatal outcomes among live births (multiple births and low birth weight), as well as biochemical pregnancy, clinical pregnancy, and miscarriage (**Table 5**). In fresh ET, live birth rates were 45.9% vs 46.8% (aRR = 0.94; 95% CI 0.78–1.14), with similar biochemical (55.6% vs 57.4%) and clinical pregnancy rates (51.2% vs 53.6%). In FET cycles, live birth rates were also comparable (39.0% vs 42.6%; aRR = 1.08; 95% CI 0.90–1.30), and other pregnancy outcomes—including miscarriage and low birth weight—showed no significant variation between sexes. Pooled comparison with NK couples (**Supplementary Table S7**; **Supplementary Figure S3**) yielded consistent findings, further supporting the absence of any detrimental reproductive impact associated with either male or female inv (9) carriage.

**Table 5:** Pregnancy outcomes by carrier sex.

Outcomes	M-inv(9) group (n, %)	F-inv(9) group (n, %)	Effect estimate (95% CI)	
			Unadjusted	Multivariable adjusted
Live birth (Primary outcome)	95/207 (45.9%)	124/265 (46.8%)	1.020 (0.838-1.240)	0.939 (0.776-1.135)^a^
Multiple births	21/95 (22.1%)	19/124 (15.3%)	0.693 (0.396-1.214)	0.515 (0.284-0.933)
Low birth weight	21/95 (22.1%)	24/124 (19.4%)	0.876 (0.520-1.475)	0.780 (0.453-1.344)
Biochemical pregnancy	115/207 (55.6%)	152/265 (57.4%)	1.032 (0.880-1.212)	0.975 (0.835-1.139)
Clinical pregnancy	106/207 (51.2%)	142/265 (53.6%)	1.046 (0.879-1.245)	0.965 (0.814-1.143)
Miscarriage	10/207 (4.8%)	17/265 (6.4%)	1.328 (0.621-2.839)	1.233 (0.576-2.641)
FET				
Live birth (Primary outcome)	103/264 (39.0%)	153/359 (42.6%)	1.092 (0.901-1.325)	1.080 (0.896-1.301)^b^
Multiple births	11/103 (10.7%)	9/153 (5.9%)	0.551 (0.237-1.282)	0.518 (0.216-1.243)
Low birth weight	19/103 (18.4%)	18/153 (11.8%)	0.638 (0.352-1.156)	0.604 (0.324-1.128)
Biochemical pregnancy	140/264 (53.0%)	206/359 (57.4%)	1.082 (0.937-1.250)	1.066 (0.929-1.224)
Clinical pregnancy	130/264 (49.2%)	194/359 (54.0%)	1.097 (0.940-1.282)	1.085 (0.935-1.261)
Miscarriage	26/264 (9.8%)	34/359 (9.5%)	0.962 (0.592-1.563)	0.970 (0.597-1.575)

Data are presented as number (percentage). Modified Poisson regression with a log link was used to estimate adjusted risk ratios (aRRs) and 95% confidence intervals (CIs) for all binary outcomes, including live birth, multiple births, low birth weight, biochemical pregnancy, clinical pregnancy, and miscarriage. Cluster-robust standard errors were applied, with clustering by patient identifier to account for within-patient correlation across multiple embryo transfer cycles. For neonatal outcomes, multiple births and low birth weight were calculated among live births, with live births as the denominator.

a Fresh cycle models were adjusted for female age, BMI, AMH, infertility duration, gravidity, parity, total gonadotropin dose, number of embryos transferred, endometrial thickness, fertilization method, infertility type and diagnosis, transfer day, and ovarian stimulation protocol

b Models for FET cycles were adjusted for female age, BMI, AMH, infertility duration, gravidity, parity, number of embryos transferred, endometrial thickness, fertilization method, type of infertility, infertility diagnosis, day of embryo transfer, and endometrial preparation regimen.

### Interaction analysis

Interaction models were applied to explore potential effect modifiers for the association between inv (9) and live birth in fresh ET cycles (**Supplementary Table S8**). Significant interactions were observed for total gonadotropin dose (aRR = 1.0003; 95% CI 1.0001–1.0006; P = 0.014), secondary infertility (aRR = 0.59; 95% CI 0.39–0.87; P = 0.008), and unknown infertility etiology (aRR = 0.42; 95% CI 0.21–0.83; P = 0.013), suggesting that the positive association between inv (9) and live birth was attenuated among couples with secondary or unexplained infertility. No other significant interactions were found for age, AMH, BMI, or other baseline variables.

## Discussion

Because inv (9) is one of the most frequently reported chromosomal variants in infertile populations, it is important to determine whether its detection leads to unnecessary anxiety or over-intervention in ART when no additional abnormalities are present. In this large retrospective cohort, couples carrying a pericentric inv (9) accounted for approximately 2.2% of all treatment cycles, and baseline characteristics were well balanced after PSM. inv (9) carriers exhibited comparable embryologic and reproductive outcomes—including oocyte yield, fertilization, and blastocyst formation rates—to those of NK couples. A slightly higher live birth rate was observed in inv (9) carriers during fresh ET cycles, whereas outcomes in FET cycles remained comparable between groups. Among inv (9) carriers, we observed no meaningful differences between male and female carriers in live birth, multiple births, low birth weight, biochemical pregnancy, clinical pregnancy, or miscarriage in either fresh or frozen cycles. Collectively, these findings support reassurance-based counseling for isolated inv (9) and do not justify additional ART interventions solely on the basis of this karyotype finding.

Chromosomal polymorphisms represent balanced structural variations of chromosomes that are frequently identified during cytogenetic testing in infertile populations. Multiple studies have indicated that these variants occur more often in individuals with infertility than in the general population, implying potential relevance to reproductive performance (4, 18, 19). Although their biological impact remains debated, growing evidence suggests possible associations between chromosomal polymorphisms and female reproductive capacity. Several investigations have documented higher rates of polymorphic variants such as 9qh+, inv (9), and 21pss/ps+ among women with unexplained or tubal infertility compared with fertile controls (5, 20, 21). Reported incidences vary widely—from approximately 3% to 8%—depending on the population studied and the diagnostic thresholds used (3–5). Chromosome 9 variants, particularly pericentric inversion and 9qh+ heterochromatic enlargement, are among the most frequently observed alterations (22, 23).

Our results align with prior research on chromosomal polymorphisms and IVF/ICSI outcomes (3, 24) while substantially expanding earlier findings. For instance, Liang et al. assessed 214 couples undergoing fresh day 2/3 embryo transfers and observed similar pregnancy and live birth rates between inv (9) and NK groups (16). However, that study was limited to early cleavage-stage transfers and lacked evaluation of blastocyst or FET outcomes, restricting generalizability. In contrast, our analysis included both fresh and frozen transfers with a substantially larger sample size and demonstrated that inv (9) carriers—whether male or female—had comparable embryologic and reproductive outcomes compared with NK couples. The modest advantage in live birth rate observed among inv (9) carriers during fresh ET cycles appeared to be modulated by gonadotropin dosage and infertility type, rather than by karyotype status itself.

Earlier reports suggested potential sex-specific effects, with female inv (9) carriers exhibiting higher normal fertilization and usable embryo rates compared with male carriers, suggesting a possible sex-specific influence on embryogenesis (15, 16). In our cohort, however, such differences were not observed; male and female carriers showed comparable fertilization, embryo development, and pregnancy outcomes. Likewise, miscarriage rates were similar between groups, contrasting with previous findings that miscarriage rates differed significantly between female and male inv (9) carriers (15, 23, 25). These inconsistencies likely reflect small sample sizes and incomplete adjustment for confounders such as ovarian response and infertility type in previous studies.

From a mechanistic standpoint, inv (9) typically involves heterochromatic regions surrounding the centromere, composed mainly of repetitive α-satellite DNA lacking coding or regulatory sequences (26–30). Such structural features mean that the inversion does not alter gene dosage or disrupt essential loci, thereby maintaining genomic stability. Cytogenetic and molecular data further show that meiotic pairing and segregation remain largely normal in inv (9) carriers, without increased risks of unbalanced gametes or embryonic aneuploidy (31, 32). Consistent with this, PGT-A analyses reveal no elevation in chromosomal rearrangements or developmental defects in embryos derived from inv (9) carriers (11). Epidemiologic evidence further supports this observation, showing comparable fertilization, pregnancy, and miscarriage rates between inv (9) carriers and individuals with normal karyotypes (16). Collectively, evidence from genomic, cytogenetic, and clinical perspectives indicates that inv (9) represents a phenotypically neutral chromosomal variant rather than a pathogenic aberration affecting fertility or embryonic development.

The strengths of this study include its unprecedented sample size, extended observation period, and comprehensive cycle-level data encompassing both fresh and frozen embryo transfers. Time-stratified propensity score matching minimized procedural and temporal confounding, while sex-stratified analyses clarified potential parental effects. Clinically, these findings provide evidence-based reassurance that inv (9) does not compromise ART or pregnancy outcomes and may help clinicians counsel patients with incidental karyotype findings. Nevertheless, several limitations should be acknowledged. Although we aimed to evaluate outcomes in couples where both partners carried inv (9), dual-carrier cases were extremely rare (n = 4) and thus excluded from comparative analyses. Additionally, a small subset of karyotype results originated from external laboratories, which could introduce minor inter-laboratory variability despite all reports meeting standardized G-banding criteria. As a retrospective single-center analysis, unmeasured confounding cannot be fully ruled out, and lifestyle or environmental factors were unavailable for adjustment. Furthermore, detailed neonatal outcomes were not consistently available, limiting full assessment of perinatal safety. To our knowledge, few studies have directly investigated the reproductive outcomes of couples with inv (9) undergoing ART, and therefore our results cannot be directly compared with those of previous reports. Finally, while our data were collected prospectively within a uniform electronic database, generalizability remains limited; multicenter or population-based studies will be needed to confirm these findings and assess potential interactions with other chromosomal variants.

## Conclusion

Across more than 40,000 ART cycles, the presence of inv (9) was not associated with adverse embryologic or reproductive outcomes. Live birth and miscarriage rates were comparable between inv (9) carriers and normal-karyotype couples, regardless of carrier sex. These findings provide strong evidence that inv (9) represents a benign chromosomal polymorphism without detrimental reproductive effects. Therefore, routine additional interventions or genetic counseling for isolated inv (9) carriers may not be necessary, allowing clinicians to reassure patients confidently during ART treatment.

## Data Availability

The original contributions presented in the study are included in the article/**Supplementary Material**. Further inquiries can be directed to the corresponding authors.
