# Supplementary material for: Effect of pericentric inversion of chromosome 9 on reproductive outcomes in assisted reproductive technology: a propensity score–matched cohort study
Source: Front Endocrinol (Lausanne). 2026 Mar 26;17:1811641. doi: 10.3389/fendo.2026.1811641 (PMC13061663; doi:10.3389/fendo.2026.1811641)
Supplement: Supplementary file 1 [file DataSheet1.docx]

**Table S1**. Comparison of baseline characteristics between couples with M-Inv(9) and F-Inv(9).

| Characteristic | M-Inv(9) = 401 | F-Inv(9) = 502 | p |
| --- | --- | --- | --- |
| Age | 34.53 ± 5.12 | 34.11 ± 5.20 | 0.223 |
| AMH (ng/mL) | 2.83 ± 2.53 | 2.84 ± 2.65 | 0.965 |
| BMI (kg/m^2^) | 22.15 ± 3.05 | 22.12 ± 3.55 | 0.897 |
| Basal E2 (pg/mL) | 51.47 ± 54.48 | 47.08 ± 70.38 | 0.291 |
| Basal FSH (mIU/mL) | 7.11 ± 3.79 | 7.27 ± 4.13 | 0.546 |
| Basal LH (mIU/mL) | 4.21 ± 4.88 | 4.11 ± 3.29 | 0.731 |
| Total Gn dose (IU) | 1719.40 ± 886.37 | 1755.65 ± 908.14 | 0.546 |
| Infertility duration (y) | 5.50 ± 4.61 | 4.74 ± 4.08 | 0.01 |
| Fertilization method (%) | |  | 0.769 |
| IVF | 292 (72.8%) | 360 (71.7%) |  |
| ICSI | 109 (27.2%) | 142 (28.3%) |  |
| Infertility type (%) |  |  | 0.032 |
| Primary | 157 (39.2%) | 161 (32.1%) |  |
| Secondary | 244 (60.8%) | 341 (67.9%) |  |
| Infertility diagnosis (%) | |  | 0.088 |
| Tubal factor | 267 (66.6%) | 310 (61.8%) |  |
| Male factor | 48 (12.0%) | 59 (11.8%) |  |
| Ovulatory | 32 (8.0%) | 49 (9.8%) |  |
| Genetic factor | 12 (3.0%) | 22 (4.4%) |  |
| RPL | 10 (2.5%) | 12 (2.4%) |  |
| Endometriosis | 5 (1.2%) | 11 (2.2%) |  |
| Unknown | 14 (3.5%) | 33 (6.6%) |  |
| Other | 13 (3.2%) | 6 (1.2%) |  |
| Ovarian stimulation protocol, No. (%) | |  | 0.411 |
| Agonist | 230 (57.4%) | 284 (56.6%) |  |
| Antagonist | 83 (20.7%) | 113 (22.5%) |  |
| Luteal-phase stimulation | 9 (2.2%) | 15 (3.0%) |  |
| PPOS | 18 (4.5%) | 29 (5.8%) |  |
| Mild Stimulation | 49 (12.2%) | 41 (8.2%) |  |
| Natural cycles | 4 (1.0%) | 9 (1.8%) |  |
| Other | 8 (2.0%) | 11 (2.2%) |  |

Continuous variables are presented as mean ± SD and categorical variables as number (percentage). Differences were compared using the t test or χ² test.

| Oocytes retrieved | NK | 10.0 ± 7.8 | Ref (1.0) |
| --- | --- | --- | --- |
|  | M-Inv(9) | 9.8 ± 7.4 | 1.006 (0.941-1.075) |
|  | F- Inv(9) | 10.0 ± 7.5 | 1.012 (0.953-1.074) |
| Mature oocytes | NK | 8.9 ± 7.0 | Ref (1.0) |
|  | M-Inv(9) | 8.6 ± 6.4 | 0.992 (0.928-1.061) |
|  | F- Inv(9) | 8.9 ± 6.9 | 1.015 (0.953-1.081) |
| Total fertilized | NK | 6.4 ± 5.3 | Ref (1.0) |
|  | M-Inv(9) | 6.1 ± 4.8 | 0.978 (0.908-1.053) |
|  | F- Inv(9) | 6.4 ± 5.5 | 1.017 (0.947-1.093) |
| Good-quality embryos | NK | 3.2 ± 3.4 | Ref (1.0) |
|  | M-Inv(9) | 2.9 ± 3.0 | 0.933 (0.835-1.043) |
|  | F- Inv(9) | 3.3 ± 3.7 | 1.062 (0.960-1.174) |
| Blastocyst cultured | NK | 8.6 ± 5.2 | Ref (1.0) |
|  | M-Inv(9) | 8.1 ± 4.7 | 0.943 (0.872-1.020) |
|  | F- Inv(9) | 8.8 ± 5.5 | 1.019 (0.945-1.098) |
| Blastocyst formation | NK | 4.9 ± 3.7 | Ref (1.0) |
|  | M-Inv(9) | 4.7 ± 3.6 | 0.937 (0.844-1.042) |
|  | F- Inv(9) | 5.1 ± 4.1 | 1.036 (0.943-1.137) |
| Mature rate (per oocyte retrieved) | NK | 87.2 ± 19.1 | Ref (1.0) |
|  | M-Inv(9) | 86.6 ± 19.5 | -0.49 (-2.92-1.94) |
|  | F- Inv(9) | 87.4 ± 17.9 | 0.004 (-1.89-1.90) |
| Fertilization rate (per oocyte retrieved) | NK | 63.5 ± 25.5 | Ref (1.0) |
|  | M-Inv(9) | 64.2 ± 24.4 | 0.23 (-2.51-2.97) |
|  | F- Inv(9) | 63.8 ± 24.5 | 0.03 (-2.63-2.69) |
| Blastocyst formation rate | NK | 55.6 ± 26.0 | Ref (1.0) |
|  | M-Inv(9) | 57.1 ± 27.5 | 0.70 (-3.23-4.62) |
|  | F- Inv(9) | 57.5 ± 26.4 | 1.38 (-2.06-4.82) |

**Table S2.** Comparison of assisted reproductive cycle outcomes among couples with NK and those with M-Inv(9) or F-Inv(9).

**Note:** Data are shown as mean ± SD. Poisson and linear regression models with cluster-robust standard errors were applied, adjusting for the covariates described in the main text.

**Table S3**. Baseline characteristics of couples with normal karyotypes and those with inversion of chromosome 9 who underwent fresh embryo transfer.

| Characteristic | Overall, n = 21661 | Before matching | | | After matching | | |
| --- | --- | --- | --- | --- | --- | --- | --- |
|  |  | NK = 21189 | Inv(9) = 472 | SMD | NK = 944 | Inv(9) = 472 | SMD |
| Age | 33.72 ± 4.96 | 33.73 ± 4.97 | 33.42 ± 4.57 | 0.064 | 33.56 ± 4.92 | 33.42 ± 4.57 | 0.029 |
| BMI (kg/m^2^) | 22.02 ± 3.12 | 22.01 ± 3.11 | 22.23 ± 3.46 | 0.064 | 22.20 ± 3.24 | 22.23 ± 3.46 | 0.008 |
| AMH (ng/mL) | 2.88 ± 2.21 | 2.88 ± 2.21 | 2.95 ± 2.29 | 0.029 | 2.87 ± 2.19 | 2.95 ± 2.29 | 0.034 |
| Infertility duration (y) | 4.86 ± 3.86 | 4.86 ± 3.85 | 5.03 ± 3.96 | 0.043 | 5.04 ± 3.97 | 5.03 ± 3.96 | 0.003 |
| Gravidity | 1.38 ± 1.46 | 1.38 ± 1.47 | 1.31 ± 1.33 | 0.052 | 1.37 ± 1.46 | 1.31 ± 1.33 | 0.044 |
| Parity | 0.28 ± 0.52 | 0.28 ± 0.52 | 0.24 ± 0.48 | 0.084 | 0.26 ± 0.50 | 0.24 ± 0.48 | 0.033 |
| Total Gn dose (IU) | 1988.6 ± 828.2 | 1990.2 ± 828.4 | 1912.8 ± 819.8 | 0.094 | 1944.6 ± 823.7 | 1912.8 ± 819.8 | 0.039 |
| No. of embryos transferred | 1.60 ± 0.51 | 1.60 ± 0.51 | 1.62 ± 0.50 | 0.034 | 1.62 ± 0.50 | 1.62 ± 0.50 | 0.004 |
| Endometrial thickness | 11.79 ± 2.55 | 11.79 ± 2.55 | 11.81 ± 2.50 | 0.009 | 11.83 ± 2.56 | 11.81 ± 2.50 | 0.006 |
| Day of transfer (%) |  |  |  | 0.061 |  |  | 0.030 |
| Day 3 | 16108 (74.4) | 15745 (74.3) | 363 (76.9) |  | 738 (78.2) | 363 (76.9) |  |
| Day 5/6 | 5553 (25.6) | 5444 (25.7) | 109 (23.1) |  | 206 (21.8) | 109 (23.1) |  |
| Fertilization method (%) | |  |  | 0.033 |  |  | 0.021 |
| IVF | 16833 (77.7) | 16460 (77.7) | 373 (79.0) |  | 738 (78.2) | 373 (79.0) |  |
| ICSI | 4828 (22.3) | 4729 (22.3) | 99 (21.0) |  | 206 (21.8) | 99 (21.0) |  |
| Infertility type (%) |  |  |  | 0.002 |  |  | 0.007 |
| Primary | 7726 (35.7) | 7558 (35.7) | 168 (35.6) |  | 333 (35.3) | 168 (35.6) |  |
| Secondary | 13935 (64.3) | 13631 (64.3) | 304 (64.4) |  | 611 (64.7) | 304 (64.4) |  |
| Infertility diagnosis (%) | |  |  | 0.102 |  |  | 0.110 |
| Tubal factor | 15088 (69.7) | 14747 (69.6) | 341 (72.2) |  | 697 (73.8) | 341 (72.2) |  |
| Male factor | 3377 (15.6) | 3312 (15.6) | 65 (13.8) |  | 136 (14.4) | 65 (13.8) |  |
| Ovulatory | 1219 (5.6) | 1188 (5.6) | 31 (6.6) |  | 56 (5.9) | 31 (6.6) |  |
| Genetic factor | 28 (0.1) | 27 (0.1) | 1 (0.2) |  | 0 (0.0) | 1 (0.2) |  |
| RPL | 3 (0.0) | 3 (0.0) | 0 (0.0) |  | 0 (0.0) | 0 (0.0) |  |
| Endometriosis | 524 (2.4) | 514 (2.4) | 10 (2.1) |  | 18 (1.9) | 10 (2.1) |  |
| Unknown | 1386 (6.4) | 1363 (6.4) | 23 (4.9) |  | 37 (3.9) | 23 (4.9) |  |
| Other | 36 (0.2) | 35 (0.2) | 1 (0.2) |  | 0 (0.0) | 1 (0.2) |  |
| Ovarian stimulation protocol, No. (%) | |  |  | 0.082 |  |  | 0.081 |
| Agonist | 15841 (73.1) | 15502 (73.2) | 339 (71.8) |  | 677 (71.7) | 339 (71.8) |  |
| Antagonist | 4476 (20.7) | 4374 (20.6) | 102 (21.6) |  | 218 (23.1) | 102 (21.6) |  |
| Mild Stimulation | 930 (4.3) | 905 (4.3) | 25 (5.3) |  | 36 (3.8) | 25 (5.3) |  |
| Natural cycles | 87 (0.4) | 85 (0.4) | 2 (0.4) |  | 3 (0.3) | 2 (0.4) |  |
| Other | 327 (1.5) | 323 (1.5) | 4 (0.8) |  | 10 (1.1) | 4 (0.8) |  |

Continuous variables are presented as mean ± SD, and categorical variables are shown as number (percentage). Data are reported before and after 1:2 PSM. The SMD was used to assess covariate balance between groups, with an absolute SMD ≥ 0.10 indicating a meaningful imbalance.

**Table S4.** Comparison of characteristics between couples with M-Inv(9) and F-Inv(9) undergoing fresh embryo transfer.

| Characteristic | M-Inv(9) = 207 | F-Inv(9) = 265 | p |
| --- | --- | --- | --- |
| Age | 33.60 ± 4.53 | 33.29 ± 4.61 | 0.461 |
| AMH (ng/mL) | 2.84 ± 2.03 | 3.03 ± 2.47 | 0.353 |
| BMI (kg/m^2^) | 21.88 ± 3.05 | 22.50 ± 3.73 | 0.0458 |
| Infertility duration (y) | 5.31 ± 4.23 | 4.80 ± 3.72 | 0.171 |
| Gravidity | 1.34 ± 1.43 | 1.28 ± 1.24 | 0.66 |
| Parity | 0.23 ± 0.47 | 0.25 ± 0.48 | 0.696 |
| Total Gn dose (IU) | 1846.61 ± 800.79 | 1964.46 ± 832.24 | 0.12 |
| No. of embryos transferred | 1.64 ± 0.51 | 1.60 ± 0.50 | 0.365 |
| Endometrial thickness | 11.83 ± 2.56 | 11.79 ± 2.47 | 0.874 |
| Day of transfer (%) |  |  | 0.108 |
| Day 3 | 167 (80.7%) | 196 (74.0%) |  |
| Day 5/6 | 40 (19.3%) | 69 (26.0%) |  |
| Fertilization method (%) | |  | 0.805 |
| IVF | 162 (78.3%) | 211 (79.6%) |  |
| ICSI | 45 (21.7%) | 54 (20.4%) |  |
| Infertility type (%) |  |  | 0.585 |
| Primary | 77 (37.2%) | 91 (34.3%) |  |
| Secondary | 130 (62.8%) | 174 (65.7%) |  |
| Infertility diagnosis (%) | |  | 0.009 |
| Tubal factor | 155 (74.9%) | 186 (70.2%) |  |
| Male factor | 34 (16.4%) | 31 (11.7%) |  |
| Ovulatory | 11 (5.3%) | 20 (7.5%) |  |
| Genetic factor | 1 (0.5%) | 0 (0.0%) |  |
| Endometriosis | 3 (1.4%) | 7 (2.6%) |  |
| Unknown | 3 (1.4%) | 20 (7.5%) |  |
| Other | 0 (0.0%) | 1 (0.4%) |  |
| Ovarian stimulation protocol, No. (%) | |  | 0.055 |
| Agonist | 150 (72.5%) | 189 (71.3%) |  |
| Antagonist | 37 (17.9%) | 65 (24.5%) |  |
| Mild Stimulation | 16 (7.7%) | 9 (3.4%) |  |
| Natural cycles | 1 (0.5%) | 1 (0.4%) |  |
| Other | 3 (1.4%) | 1 (0.4%) |  |

Continuous variables are presented as mean ± SD, and categorical variables as number (percentage). p-values were calculated using t-tests for continuous variables and χ² tests for categorical variables to compare couples with male M-Inv(9) and F-Inv(9) undergoing fresh embryo transfer.

**Table S5.** Baseline characteristics of couples with normal karyotypes and those with inversion of chromosome 9 who underwent frozen embryo transfer.

| Characteristic | Overall, n = 28522 | Before matching | | | After matching | | |
| --- | --- | --- | --- | --- | --- | --- | --- |
|  |  | NK = 27899 | Inv(9) = 623 | SMD | NK = 1246 | Inv(9) = 623 | SMD |
| Age | 34.06 ± 4.98 | 34.06 ± 4.99 | 34.09 ± 4.80 | 0.007 | 33.98 ± 5.02 | 34.09 ± 4.80 | 0.024 |
| BMI (kg/m^2^) | 21.81 ± 3.09 | 21.80 ± 3.08 | 22.05 ± 3.27 | 0.080 | 22.11 ± 3.20 | 22.05 ± 3.27 | 0.016 |
| AMH (ng/mL) | 3.41 ± 3.07 | 3.41 ± 3.08 | 3.45 ± 2.79 | 0.013 | 3.45 ± 2.68 | 3.45 ± 2.79 | 0.002 |
| Infertility duration (y) | 4.89 ± 3.82 | 4.89 ± 3.81 | 5.22 ± 4.37 | 0.082 | 5.12 ± 4.02 | 5.22 ± 4.37 | 0.023 |
| Gravidity | 1.25 ± 1.40 | 1.25 ± 1.40 | 1.19 ± 1.33 | 0.045 | 1.20 ± 1.30 | 1.19 ± 1.33 | 0.010 |
| Parity | 0.22 ± 0.47 | 0.22 ± 0.47 | 0.20 ± 0.44 | 0.036 | 0.21 ± 0.47 | 0.20 ± 0.44 | 0.021 |
| No. of embryos transferred | 1.43 ± 0.52 | 1.43 ± 0.52 | 1.43 ± 0.51 | 0.015 | 1.45 ± 0.51 | 1.43 ± 0.51 | 0.022 |
| Endometrial thickness | 10.48 ± 2.00 | 10.47 ± 2.00 | 10.50 ± 1.99 | 0.014 | 10.53 ± 2.02 | 10.50 ± 1.99 | 0.016 |
| Fertilization method (%) | |  |  | 0.028 |  |  | 0.058 |
| IVF | 22295 (78.2) | 21801 (78.1) | 494 (79.3) |  | 958 (76.9) | 494 (79.3) |  |
| ICSI | 6227 (21.8) | 6098 (21.9) | 129 (20.7) |  | 288 (23.1) | 129 (20.7) |  |
| Infertility type (%) |  |  |  | 0.003 |  |  | 0.023 |
| Primary | 11163 (39.1) | 10920 (39.1) | 243 (39.0) |  | 472 (37.9) | 243 (39.0) |  |
| Secondary | 17359 (60.9) | 16979 (60.9) | 380 (61.0) |  | 774 (62.1) | 380 (61.0) |  |
| Day of transfer (%) |  |  |  | 0.004 |  |  | 0.010 |
| Day 3 | 9440 (33.1) | 9235 (33.1) | 205 (32.9) |  | 404 (32.4) | 205 (32.9) |  |
| Day 5/6 | 19082 (66.9) | 18664 (66.9) | 418 (67.1) |  | 842 (67.6) | 418 (67.1) |  |
| Endometrial preparation, No. (%) | |  |  | 0.096 |  |  | 0.022 |
| Natural cycle | 7952 (27.9) | 7804 (28.0) | 148 (23.8) |  | 308 (24.7) | 148 (23.8) |  |
| Programmed cycle | 20570 (72.1) | 20095 (72.0) | 475 (76.2) |  | 938 (75.3) | 475 (76.2) |  |

Continuous variables are presented as mean ± SD, and categorical variables are shown as number (percentage). Data are reported before and after 1:2 PSM.

**Table S6.** Comparison of characteristics between couples with M-Inv(9) and F-Inv(9) undergoing FET.

| Characteristic | M-Inv(9) = 264 | F-Inv(9) = 359 | p |
| --- | --- | --- | --- |
| Age | 34.27 ± 4.91 | 33.96 ± 4.72 | 0.432 |
| AMH (ng/mL) | 3.34 ± 2.71 | 3.53 ± 2.86 | 0.414 |
| BMI (kg/m^2^) | 22.39 ± 3.23 | 21.81 ± 3.29 | 0.0275 |
| Infertility duration (y) | 5.67 ± 4.83 | 4.89 ± 3.97 | 0.0326 |
| Gravidity | 1.27 ± 1.44 | 1.13 ± 1.25 | 0.225 |
| Parity | 0.22 ± 0.46 | 0.18 ± 0.42 | 0.272 |
| No. of embryos transferred | 1.44 ± 0.50 | 1.43 ± 0.51 | 0.979 |
| Endometrial thickness | 10.61 ± 2.08 | 10.42 ± 1.92 | 0.239 |
| Fertilization method (%) | |  | 0.301 |
| IVF | 215 (81.4%) | 279 (77.7%) |  |
| ICSI | 49 (18.6%) | 80 (22.3%) |  |
| Infertility type (%) |  |  | 0.564 |
| Primary | 99 (37.5%) | 144 (40.1%) |  |
| Secondary | 165 (62.5%) | 215 (59.9%) |  |
| Day of transfer (%) |  |  | 0.188 |
| Day 3 | 95 (36.0%) | 110 (30.6%) |  |
| Day 5/6 | 169 (64.0%) | 249 (69.4%) |  |
| Endometrial preparation, No. (%) | |  | 0.967 |
| Natural cycle | 62 (23.5%) | 86 (24.0%) |  |
| Programmed cycle | 202 (76.5%) | 273 (76.0%) |  |

Continuous variables are presented as mean ± SD, and categorical variables as number (percentage). P-values were calculated using t-tests for continuous variables and χ² tests for categorical variables to compare M-Inv(9) and F-Inv(9) couples undergoing FET.

**Table S7.** Comparison of pregnancy outcomes between couples with NK and those with M-Inv(9), F-Inv(9) in fresh and frozen embryo transfer cycles.

| Fresh embryo transfer | Group | n, % | Adjusted effect (95% CI) |
| --- | --- | --- | --- |
| Live birth (Primary outcome) | NK | 389 (41.2%) | 1 [Reference] |
|  | M-Inv(9) | 95 (45.9%) | 1.171 (0.996-1.378) |
|  | F- Inv(9) | 124 (46.8%) | 1.100 (0.957-1.264) |
| Multiple births | NK | 66 (17.0%) | 1 [Reference] |
|  | M-Inv(9) | 21 (22.1%) | 1.303 (0.842-2.017) |
|  | F- Inv(9) | 19 (15.3%) | 0.903 (0.565-1.443) |
| Low birth weight | NK | 70 (18.0%) | 1 [Reference] |
|  | M-Inv(9) | 21 (22.1%) | 1.228 (0.797-1.894) |
|  | F- Inv(9) | 24 (19.4%) | 1.076 (0.709-1.633) |
| Biochemical pregnancy | NK | 519 (55.0%) | 1 [Reference] |
|  | M-Inv(9) | 115 (55.6%) | 1.049 (0.920-1.196) |
|  | F- Inv(9) | 152 (57.4%) | 1.016 (0.908-1.137) |
| Clinical pregnancy | NK | 476 (50.4%) | 1 [Reference] |
|  | M-Inv(9) | 106 (51.2%) | 1.060 (0.918-1.224) |
|  | F- Inv(9) | 142 (53.6%) | 1.032 (0.915-1.165) |
| Miscarriage | NK | 75 (7.9%) | 1 [Reference] |
|  | M-Inv(9) | 10 (4.8%) | 0.632 (0.332-1.205) |
|  | F- Inv(9) | 17 (6.4%) | 0.807 (0.483-1.349) |
| FET | | | |
| Live birth (Primary outcome) | NK | 499 (40.0%) | 1 [Reference] |
|  | M-Inv(9) | 103 (39.0%) | 0.998 (0.843-1.180) |
|  | F- Inv(9) | 153 (42.6%) | 1.078 (0.944-1.231) |
| Multiple births | NK | 62 (12.4%) | 1 [Reference] |
|  | M-Inv(9) | 11 (10.7%) | 0.860 (0.469-1.575) |
|  | F- Inv(9) | 9 (5.9%) | 0.473 (0.241-0.930) |
| Low birth weight | NK | 70 (14.0%) | 1 [Reference] |
|  | M-Inv(9) | 19 (18.4%) | 1.315 (0.830-2.084) |
|  | F- Inv(9) | 18 (11.8%) | 0.839 (0.516-1.363) |
| Biochemical pregnancy | NK | 683 (54.8%) | 1 [Reference] |
|  | M-Inv(9) | 140 (53.0%) | 0.990 (0.869-1.128) |
|  | F- Inv(9) | 206 (57.4%) | 1.052 (0.951-1.163) |
| Clinical pregnancy | NK | 621 (49.8%) | 1 [Reference] |
|  | M-Inv(9) | 130 (49.2%) | 1.012 (0.878-1.167) |
|  | F- Inv(9) | 194 (54.0%) | 1.092 (0.978-1.219) |
| Miscarriage | NK | 108 (8.7%) | 1 [Reference] |
|  | M-Inv(9) | 26 (9.8%) | 1.172 (0.768-1.789) |
|  | F- Inv(9) | 34 (9.5%) | 1.094 (0.747-1.602) |

Data are presented as number (percentage). Adjusted effect estimates were obtained using modified Poisson regression with a log link and cluster-robust standard errors (clustered by patient ID) for all binary outcomes, including live birth, multiple births, low birth weight, biochemical pregnancy, clinical pregnancy, and miscarriage, reporting adjusted relative risks (aRRs) and 95% confidence intervals (CIs). For neonatal outcomes, multiple births and low birth weight were calculated among live births, with live births as the denominator. All models were adjusted for relevant covariates, as specified for fresh and frozen embryo transfer cycles.

**Table S8.** Interaction analysis between chromosome 9 inversion status and baseline characteristics for live birth in fresh embryo transfer cycles.

| **Interaction** | **live birth, aRR (95% CI)** | **p** |
| --- | --- | --- |
| Inv(9): age | 1.00 (0.96-1.04) | 0.8829 |
| Inv(9): AMH | 0.98 (0.90-1.07) | 0.6152 |
| Inv(9): BMI | 0.98 (0.92-1.03) | 0.3931 |
| Inv(9): Infertility duration | 1.03 (0.97-1.09) | 0.3812 |
| Inv(9): gravidity | 0.90 (0.77-1.05) | 0.1868 |
| Inv(9): parity | 0.69 (0.46-1.05) | 0.0817 |
| Inv(9): No. of embryos transferred | 1.33 (0.90-1.96) | 0.1466 |
| Inv(9): Total Gn dose | **1.0003 (1.0001-1.0006)** | **0.014** |
| Inv(9): Endometrial thickness | 1.01 (0.93-1.10) | 0.8215 |
| Inv(9): Secondary infertility | **0.59 (0.39-0.87)** | **0.0079** |
| Inv(9): ICSI | 0.72 (0.43-1.21) | 0.2106 |
| Inv(9): Day 5/6 | 0.86 (0.56-1.34) | 0.5104 |
| Inv(9): Male factor | 0.94 (0.57-1.53) | 0.7971 |
| Inv(9): Ovulatory | 1.44 (0.53-3.88) | 0.4698 |
| Inv(9): Endometriosis | 1.02 (0.38-2.72) | 0.9659 |
| Inv(9): Unknown | **0.42 (0.21-0.83)** | **0.0125** |
| Inv(9): Antagonist | 0.78 (0.49-1.26) | 0.3128 |
| Inv(9): Mild Stimulation | 0.46 (0.04-5.02) | 0.5245 |
| Inv(9): other | 2.95 (0.44-19.82) | 0.266 |

Interaction effects between 9 inversion couples and baseline characteristics on live birth outcomes were estimated using Poisson regression with robust standard errors clustered by patient ID. Models included 9 inversion status, each baseline variable, and their interaction term. Results are shown as aRR with 95% CIs. P values correspond to the interaction term. p < 0.05 was considered statistically significant.
