# Supplementary material for: Effect of pericentric inversion of chromosome 9 on reproductive outcomes in assisted reproductive technology: a propensity score–matched cohort study
Source: Front Endocrinol (Lausanne). 2026 Mar 26;17:1811641. doi: 10.3389/fendo.2026.1811641 (PMC13061663; doi:10.3389/fendo.2026.1811641)
Supplement: Supplementary file 2 [file DataSheet2.docx]

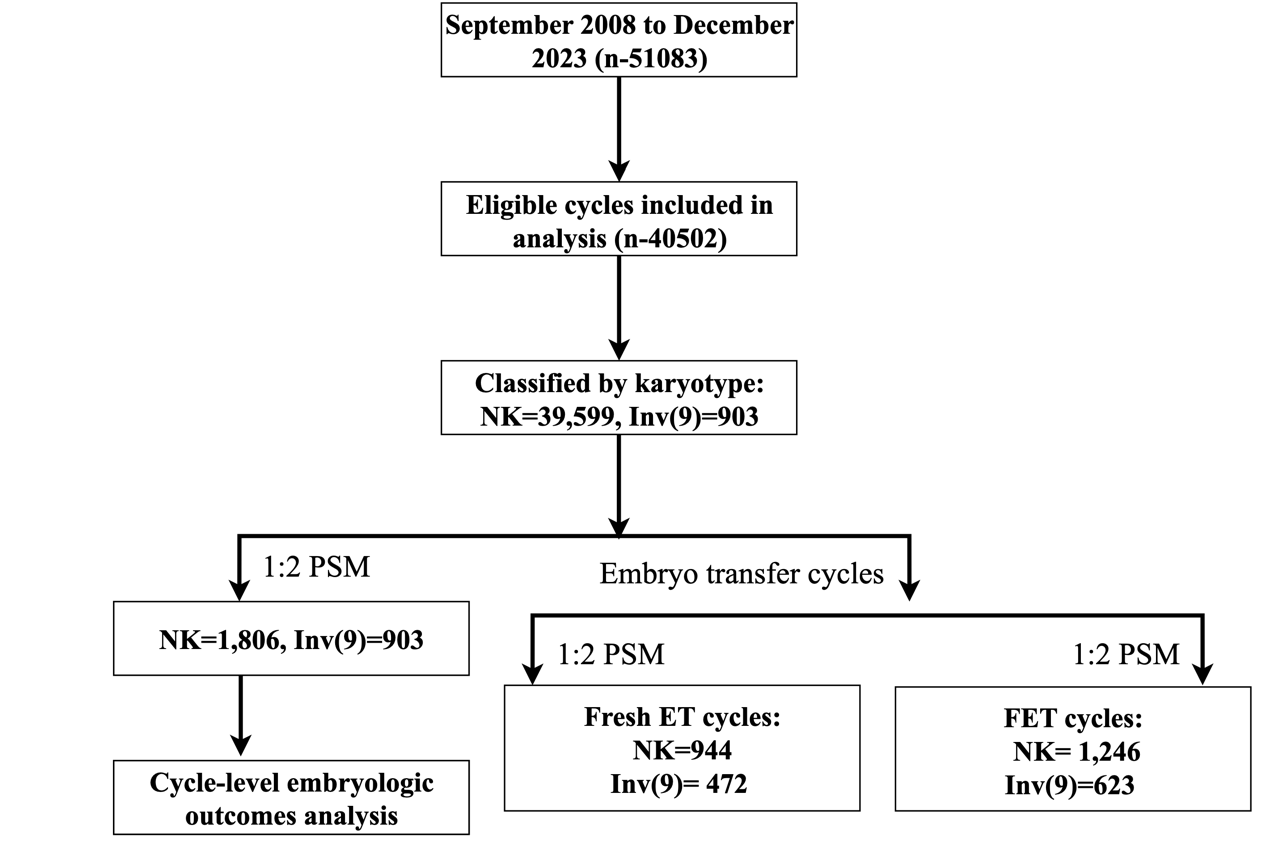


**Figure S1.** Flow diagram of study population selection and propensity score matching.


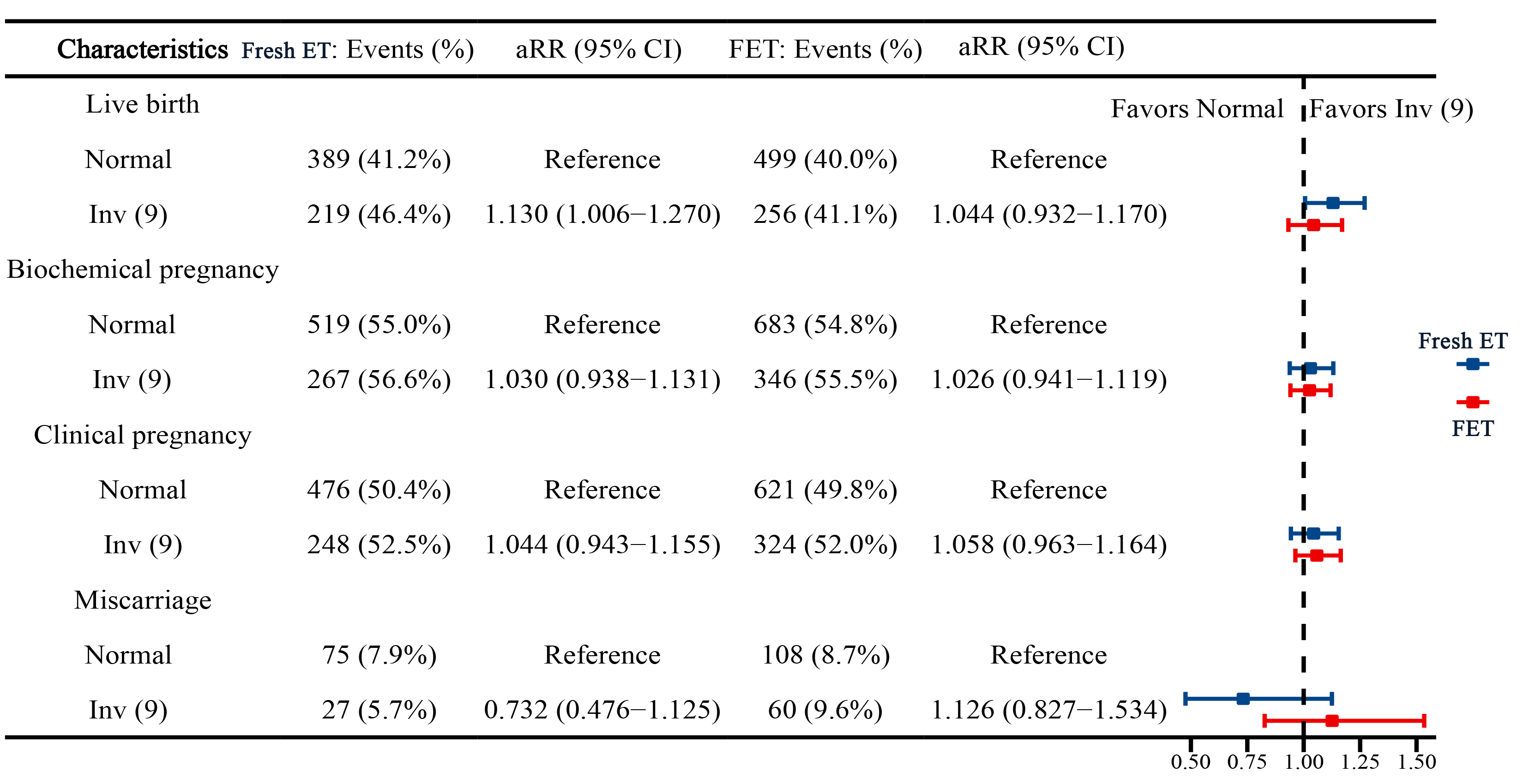


**Figure S2.** Forest plot of aRRs and 95% CIs for pregnancy outcomes comparing couples with inversion 9 and normal karyotypes in fresh and frozen embryo transfer cycles.

Modified Poisson regression with cluster-robust standard errors was used.


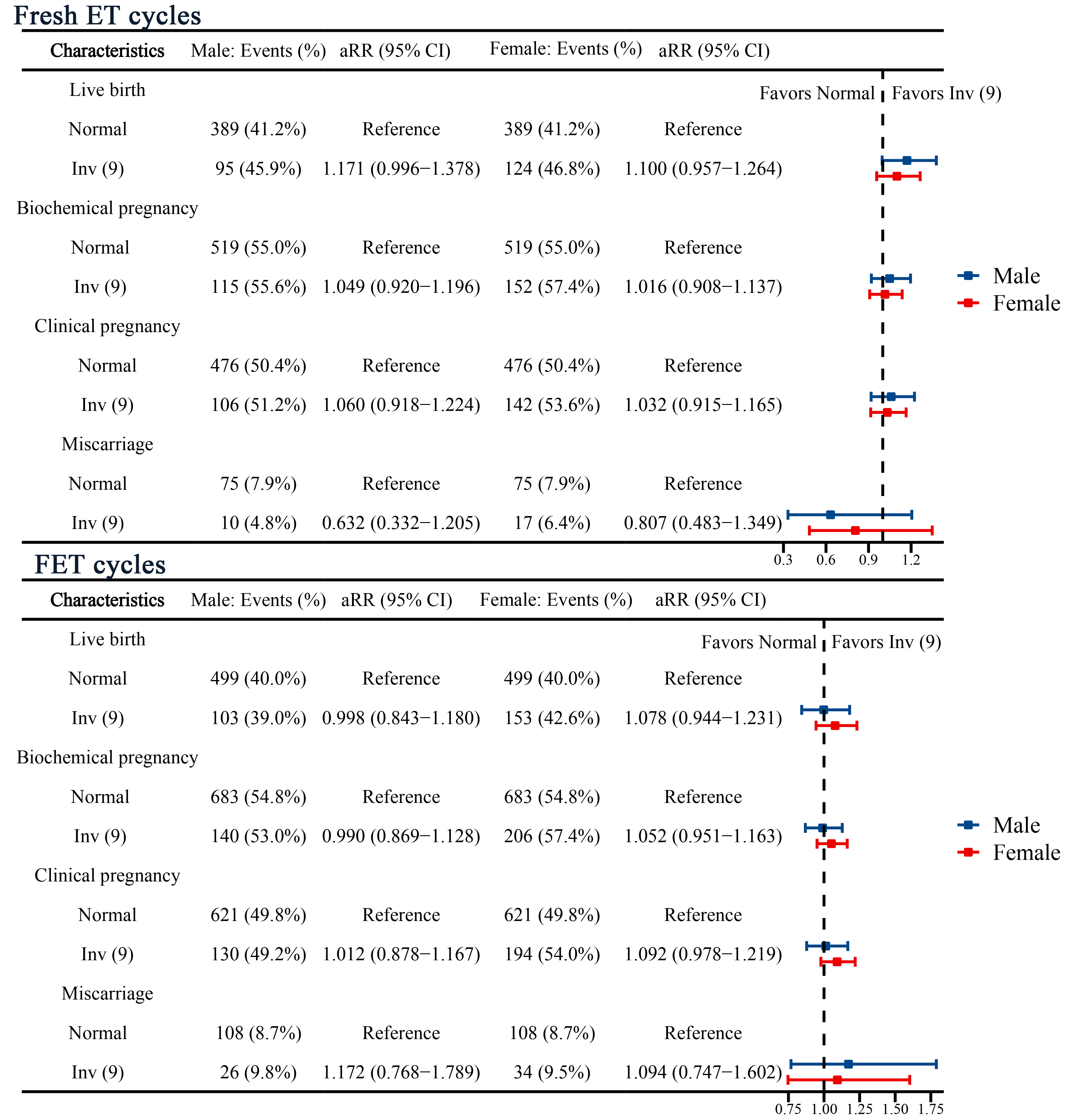


**Figure S3.** Forest plot comparing pregnancy outcomes between couples with normal karyotypes and those with M-Inv (9) or F-Inv (9) in fresh and frozen embryo transfer cycles. Data are presented as aRR with 95% CIs. Modified Poisson regression with cluster-robust standard errors were used. Blue represents M-Inv (9), and Red represents F-Inv (9) in the forest plot.
